# Bottom-Up Proteomics: Advancements in Sample Preparation

**DOI:** 10.3390/ijms24065350

**Published:** 2023-03-10

**Authors:** Van-An Duong, Hookeun Lee

**Affiliations:** College of Pharmacy, Gachon University, Incheon 21936, Republic of Korea; anduong@gachon.ac.kr

**Keywords:** proteomics, sample preparation, in-solution digestion, FASP, S-Trap, SP3, LC-MS/MS, automation

## Abstract

Liquid chromatography–tandem mass spectrometry (LC–MS/MS)-based proteomics is a powerful technique for profiling proteomes of cells, tissues, and body fluids. Typical bottom-up proteomic workflows consist of the following three major steps: sample preparation, LC–MS/MS analysis, and data analysis. LC–MS/MS and data analysis techniques have been intensively developed, whereas sample preparation, a laborious process, remains a difficult task and the main challenge in different applications. Sample preparation is a crucial stage that affects the overall efficiency of a proteomic study; however, it is prone to errors and has low reproducibility and throughput. In-solution digestion and filter-aided sample preparation are the typical and widely used methods. In the past decade, novel methods to improve and facilitate the entire sample preparation process or integrate sample preparation and fractionation have been reported to reduce time, increase throughput, and improve reproducibility. In this review, we have outlined the current methods used for sample preparation in proteomics, including on-membrane digestion, bead-based digestion, immobilized enzymatic digestion, and suspension trapping. Additionally, we have summarized and discussed current devices and methods for integrating different steps of sample preparation and peptide fractionation.

## 1. Introduction

Proteomics is an analytical technique that examines protein expression, structures, functions, and interactions in a particular cell, tissue, body fluid, or organism [1,2]. Protein composition and abundance are currently analyzed to identify disease markers or treatment mechanisms, as all changes in proteomes indicate pathological or biological processes [3,4,5]. Over the last two decades, liquid chromatography–tandem mass spectrometry (LC-MS/MS)-based proteomics has been developed as an alternative to time-consuming and labor-intensive gel-based proteomics and immunoassays [6]. LC–MS/MS-based proteomics has high throughput because thousands of peptides and proteins can be analyzed in a short time [7]. Furthermore, it can be automated to improve high-throughput performance, precision, and repeatability [8]. Some liquid-handling workstations, such as Agilent AssayMAP Bravo [9] and Biomek NXP Span-8 [10], can perform most steps of proteomic workflows.

The two analytical procedures frequently used in proteomics are top-down and bottom-up approaches. In top-down proteomics, intact proteins are directly separated and analyzed using LC–MS/MS to identify, characterize, and quantify proteoforms (distinct proteins generated from a particular gene owing to genetic variations), alternative RNA splicing, and post-translational modifications (PTMs) [11,12,13]. Conversely, in bottom-up proteomics, proteins undergo enzymatic proteolysis and the resultant peptides are analyzed and identified. This strategy has been widely used because peptides are easier to separate and identify than proteins [14]. The peptide mixture in bottom-up proteomics comprises thousands of peptides; thus, multidimensional separation is usually performed for in-depth proteome analysis [15].

A typical LC–MS/MS-based proteomic workflow consists of three major steps: sample preparation, protein/peptide separation coupled with MS/MS analysis, and data analysis [16,17]. The two proteomic approaches differ mainly in sample preparation. Top-down proteomics include protein extraction from biological samples and sample purification [18]. However, bottom-up proteomics require additional steps including protein reduction, alkylation, and enzymatic digestion [14]. Each stage of the bottom-up proteomic workflow has tremendously developed over the last two decades. The development of high-performance liquid chromatography (HPLC) instrumentation over the past decade has facilitated proteomic research by simultaneously separating many peptides in a single run [19,20]. Reversed-phase liquid chromatography (RPLC) plays a critical role in protein/peptide separation prior to MS/MS analysis [21]. Other separation mechanisms, such as strong cation exchange chromatography (SCX), strong anion exchange chromatography (SAX), size exclusion chromatography (SEC), and hydrophilic interaction chromatography (HILIC), are frequently combined with RPLC to develop multidimensional separation platforms [15]. Advances in multidimensional separation of proteins/peptides have been demonstrated in various systems, including SCX–RPLC [22,23], SAX–RPLC [24], SEC–RPLC [25,26], HILIC–RPLC [27,28], RPLC–RPLC [29,30], SCX–RPLC–RPLC [31,32,33], SAX–RPLC–RPLC [34], SCX–HILIC–RPLC [35], and RPLC–RPLC–RPLC [36].

The mass spectrometer has also been improved in the past decade with the development of new fragmentation techniques and substantial increases in scan speed and mass accuracy [37,38,39], enabling the first profiling of the human proteome draft in 2014 [37,38]. Some types of mass analyzers frequently used in proteomics are quadrupole, ion-trap, time-of-flight, orbitrap, and Fourier-transform ion cyclotron resonance [39,40]. In recent proteomic studies, data have been analyzed using high-throughput and time-efficient software [41]. Raw MS/MS data are searched against databases to identify and quantify peptides and proteins using search engines such as X!Tandem [42], Mascot [43], Sequest [44], Comet [45], Maxquant [46], Byonic [47], MSFragger [48], and Open-pFind [49]. The results are further subjected to statistical tests to identify differentially expressed proteins (DEPs), enrichment analysis to determine biological relevance, and network analysis to visualize protein–protein interactions and protein groups [41].

**Figure 1:**
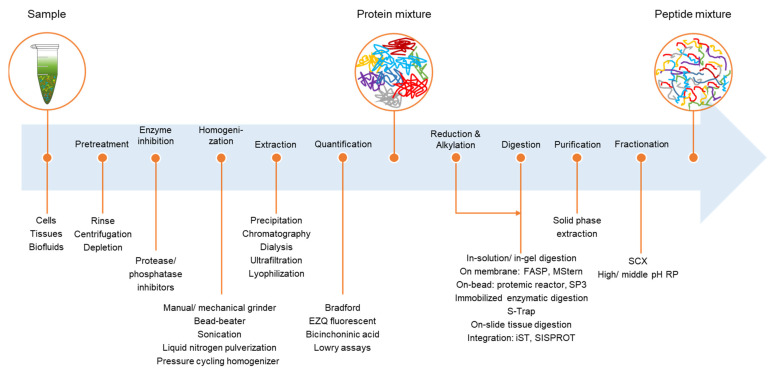
Sample preparation in bottom-up proteomics.

Apart from these stages, sample preparation remains a difficult task and the main challenge of bottom-up proteomics with laborious steps [50]. Generally, sample preparation aims to create a less complex peptide mixture that is suitable for analysis. It requires pre-fractionation; depletion of most unnecessarily abundant proteins; removal of DNA, lipids, and small metabolites; and sample clean-up from impurities (salts and remaining solid particles) [51]. Therefore, typical sample preparation processes for bottom-up proteomics usually include lysis/homogenization, protein extraction/precipitation, reduction, alkylation, enzymatic digestion, fractionation, and desalting (Figure 1). Sample preparation is an essential stage affecting the overall efficiency of proteomic studies. However, it is error-prone and has low reproducibility and throughput [52]. The early stages of proteomics used in-gel sample preparation [53]. Gel-free sample preparation has been developed in the past decade and is widely used in proteomic studies. Typical strategies include in-solution digestion (ISD), filter-aided sample preparation (FASP) [54], suspension trapping (S-Trap) [55], and single-pot solid-phase-enhanced sample preparation (SP3) [56]. Many groups have reported novel methods to improve and facilitate the entire sample preparation process or some of its steps to reduce time, increase throughput, and improve reproducibility. Single-cell proteomics has been developed in the past decades to handle samples with low protein amounts [57]. Typical single-cell proteomic approaches are: nanodroplet processing in one pot for a trace sample (nanoPOTS) [58]; nanoliter-scale oil-air-droplet (OAD) chip [59]; an integrated device for single-cell analysis (iPAD-1) [60]; digital microfluidic isolation of single cells for -omics (DISCO) [61]; and integrated spectral library-based single-cell proteomics [62]. However, this review does not cover single-cell proteomics due to their unique characteristics. In this review, we have summarized the current methods for preparing different proteomic sample types. In addition, we have presented and discussed recent developments to enhance the sample preparation process and integrate different sample preparation steps as well as their applications to biological samples.

## 2. From Biological Samples to Proteins

The first stage of a bottom-up proteomic workflow is to obtain a protein mixture from biological samples. This includes sample pretreatment, enzyme inhibition, homogenization, protein extraction/precipitation, and protein fractionation. The procedures vary depending on the sample type. They are also used for sample preparation in top-down proteomics.

### 2.1. Cells

For cell samples, it is necessary to break down the cell membranes and homogenize the samples with lysis buffer and sonication [63]. During this process, some enzymes that may affect the protein structure, such as proteases and phosphatases, are released. Therefore, these enzymes should be inhibited by maintaining the samples at a low temperature and adding an enzyme inhibitor cocktail. Typical protease inhibitors include pepstatin A, leupeptin, aprotinin, and chymostatin [64]. They are commercially available independently or in mixtures. Phosphatase inhibitors, including sodium fluoride, sodium orthovanadate, sodium pyrophosphate, and beta-glycerophosphate, are used to protect phosphorylated proteins [65]. These enzyme inhibitors are usually added to the lysis buffers prior to sonication.

Homogenized samples are then subjected to a protein extraction step to obtain samples with high protein concentrations before digestion. Protein precipitation is the most common method for protein extraction, particularly for diluted samples. Organic solvents (such as acetone, methanol, or ethanol) and their mixtures with trichloroacetic acid or sodium deoxycholate are generally used to precipitate proteins. The protein pellets are then collected and washed with pre-chilled solvent to remove contaminants [66,67]. Protein precipitation with acetone is widely used because it rapidly dissolves non-polar contaminants (such as lipids). In addition, chromatography, electrophoresis, dialysis, ultrafiltration, lyophilization, and crystallization have also been used for protein purification [68].

### 2.2. Biological Fluids

Biological fluids (blood, urine, saliva, nasal fluids, tears, and aqueous humor) need to be diluted with an appropriate amount of buffer to facilitate the subsequent enzymatic digestion process [69,70]. In the case of urine samples, proteins are extracted using solvent precipitation, ultrafiltration, centrifugal filtration, dialysis, and lyophilization [71]. Previously, Jesus et al. developed an ultrasonic-based, membrane-aided sample preparation for urine proteomic analysis, which includes urine filtration through a membrane (to retain proteins and remove salts) and ultrasonic energy-mediated tryptic digestion in the membrane [72].

Blood samples are usually centrifuged to collect plasma or serum. Plasma and serum are generally subjected to immunodepletion to remove high-abundance proteins, thereby allowing the identification of low-abundance proteins [73]. Approximately 10,000 proteins are present in plasma, ranging from albumin at 35 to 50 mg/mL to low-abundance proteins at pg/mL [74]. Immunodepletion (spin columns and LC) and magnetic beads can remove up to the 20 most abundant plasma proteins [75,76,77,78]. Depletion results in carry-over, low reproducibility, low throughput, and loss of many albumin-bound proteins [79]. Other fluids are centrifuged to remove debris before protein extraction [80].

### 2.3. Tissues

Tissue samples are usually rinsed with ice-cold saline to remove blood, serum, and fat [81]. Subsequently, they are homogenized using different apparatuses.

Manual tissue homogenization involves the use of a pestle and mortar. Pestles and mortars can be made of glass, glass teflon, or stainless steel. Glass mortars and pestles can generate heat, whereas glass Teflon mortars and pestles (with a nonstick Teflon surface) minimize heat generation and sample loss. The apparatus is particularly suitable for soft tissues, such as the brain and liver. After placing the tissues in a tube on ice (such as a Potter homogenizer) with lysis buffer, a pestle is used to manually homogenize the tissues until no large tissue pieces are observed [82]. Under the mechanical shear force, proteins and other molecules are released into the buffers. Mechanical rotor–stator grinders are used to vigorously mix, accelerate, and press samples through the narrow gap between the rotor and stator. They can be used for most tissue types, from soft to tough tissues [83]. Bead-beating homogenization is a flexible and efficient method for preparing soft or hard tissue in seconds. This involves shaking tissues with tungsten carbide, stainless steel, or glass beads in pre-chilled tubes [84,85]. Commercially available bead-beaters can process 4–24 samples simultaneously [86]. Sonication is effective for homogenizing soft tissues. It is typically equipped with a tip generator that causes cavitation effects that disrupt the tissue [87].

Liquid nitrogen pulverization involves the use of liquid nitrogen to freeze tissues for a short time. It can preserve protein integrity without generating heat [88]. The frozen tissues are then disrupted using a pestle and mortar [89]. A pulverizer (such as Covaris CP02 cryoPrep automated dry pulverizer) can be used to disrupt the tissues placed in strong and flexible plastic tissue tubes [90]. The resultant powder is mixed with lysis buffer containing enzyme inhibitors and lysed. This method is applicable to all types of tissues, particularly hard tissues and those with tough connective fibers.

Pressure cycling homogenizer (PCT) is a technique that involves the use of hydrostatic pressure and mechanical grinding. The instrument (Barocycler) compresses air to create high pressure inside the reaction chamber. The pressure rapidly increased from ambient pressure to ~45,000 psi over several cycles, resulting in tissue disruption. This method is only applicable to soft tissues, whereas some hard and large tissues remain incompletely lysed [91].

Similar to cells, homogenized tissues can be subjected to a protein extraction step to obtain samples with high protein concentrations.

### 2.4. Protein Quantification

Protein concentrations in the samples are determined using different methods before protein digestion. Colorimetric dye methods are based on the color change upon protein–dye binding, whereas fluorescent dye methods rely on the fluorescence associated with the dye after protein–dye binding. Colorimetric dye methods (such as Bradford assay) are straightforward, fast, and compatible with most solvents, buffers, reducing substances, salts, thiols, and metal-chelating agents [92]. Fluorescent dye methods (such as EZQ fluorescent and Qubit protein assays) are susceptible and suitable for low protein samples [93]. Biuret methods rely on protein–copper chelation in an alkaline environment to reduce Cu^2+^ to Cu^+^, which reacts with a reagent in a bicinchoninic acid assay to form a purple complex that is detected between 550 and 570 nm [94]. In Lowry assays, Cu^+^ reacts with Folin–Ciocalteu reagent containing phosphotungstic acid and phosphomolybdic acid [95]. Biuret methods are compatible with most detergents and are less likely to cause protein-to-protein variations than Bradford assays [94].

## 3. From Proteins to Peptides

Sample preparation from proteins to peptides includes various steps, such as detergent removal, buffer exchange, reduction, alkylation, digestion, optional peptide fractionation, and desalting. The major bottleneck among these procedures is protein cleanup and digestion, which considerably affects the accuracy and reproducibility of protein quantification.

### 3.1. Protein Digestion

#### 3.1.1. In-Solution and In-Gel Digestion

ISD and in-gel digestion have been used since the beginning of proteomics research. The ISD can handle 100–1000 µg protein. Urea and thiourea are generally used to solubilize proteins and denature their three-dimensional structures. Proteins in 8 M urea solution are reduced and alkylated to reduce intra- and intermolecular disulfide bonds within and between protein molecules [96]. Typical reduction reagents include dithiothreitol, tris(2-carboxyethyl)phosphine (TCEP), tris(3-hydroxypropyl) phosphine, and 2-mercaptoethanol. The free sulfhydryl groups formed by reduction are alkylated with alkylation agents (iodoacetamide, iodoacetic acid, N-ethylmaleimide, and S-methyl methanethiosulfonate) to prevent disulfide bond reformation. The protein samples are then diluted to reduce the urea concentration before enzyme addition. Typical digestion enzymes are trypsin and LysC. The trypsin:protein ratio usually varies from 1:20 to 1:50. Trypsin cleavage sites are located at the amino acid residues arginine and lysine. The obtained peptides obtained after digestion are 800–2000 Da, which is appropriate for MS/MS sequencing [97]. ISD is associated with inevitable sample loss and cannot be used for detergent-containing samples. It requires a large starting amount of protein (typically > 100 µg) and long digestion time (overnight).

A modification of ISD, which can be applied to samples containing sodium dodecyl sulfate (SDS), has been developed recently [98]. This is a simple, robust, and reproducible SDS–cyclodextrin-assisted sample preparation (SCASP) method that can be applied to different sample types. After reduction and alkylation, the samples are mixed with cyclodextrin solution. SDS molecules are quickly incorporated into the internal cavities of cyclodextrins. Trypsin is then added for digestion. The SDS–cyclodextrin complex is removed from the peptide mixtures during desalting. When SCASP was used to analyze 5000 HeLa cell samples, ~2500 proteins were identified. Another ISD modification combines lysis, reduction, and alkylation into a single step to reduce time and sample loss. It is performed using the reducing agent TCEP and alkylating agent 2-chloroacetamide (CAA), which are compatible and can be directly incorporated into the lysis buffer [99,100].

Doellinger et al. have recently developed sample preparation by easy extraction and digestion (SPEED) [101]. The main feature of SPEED is the use of pure trifluoroacetic acid (TFA; pKa = 0.2) to dissolve cells and tissues within a few minutes and form clear lysates. After neutralization with a weak base (Tris(hydroxymethyl)-aminomethane, pKa = 8.1), the lysates become slightly turbid due to protein precipitation as fine particles. The proteins are then simultaneously reduced and alkylated using TCEP and CAA, respectively. Then, the samples are heated up to 70–80 °C to shorten the incubation time to 5 min. Subsequently, tryptic digestion is performed similar to that in ISD. Using SPEED, the authors identified ~2700 and ~1900 proteins in *Escherichia coli* and *Staphylococcus aureus*, respectively, which were higher than those reported in previous studies [101]. The authors demonstrated that the SPEED was highly reproducible and performed better than FASP, ISD, and SP3 for quantitative proteomics [101]. These results suggest that SPEED is a potential method for future studies in bottom-up proteomics. However, further studies are needed to evaluate this method.

In-gel digestion is time-consuming and more laborious than ISD. Proteins are first separated by one- or two-dimensional polyacrylamide gel electrophoresis. Subsequently, the gel spots containing the proteins of interest are collected, cut into small pieces (approximately 1 × 1 mm^2^), and destained with 100 mM ammonium bicarbonate/acetonitrile (1:1, *v*/*v*). The proteins are then reduced, alkylated, and digested. Peptides are usually extracted from gel pieces using 50% acetonitrile/5% formic acid [53]. ISD and in-gel digestion are less efficient, particularly for clinical samples, which require high sample processing throughput and reproducibility. Therefore, other methods have been developed to overcome these limitations.

#### 3.1.2. On-Membrane Digestion: FASP and MStern

**Figure 2:**
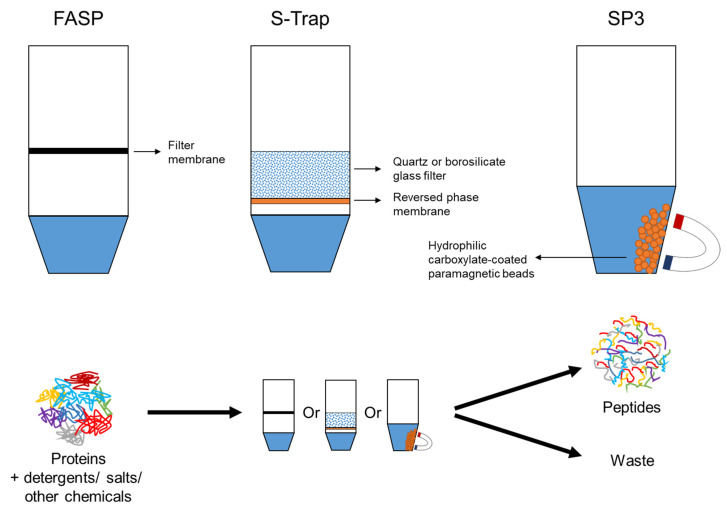
Sample preparation with FASP, S-Trap, and SP3 in bottom-up proteomics.

These methods utilize membranes to separate proteins from detergents and other small molecular impurities. The FASP was developed in 2005 by Liebler et al. [102] and in 2009 by Mann et al. [54]. This method has been widely used for bottom-up proteomic sample preparation over the past decade. All sample preparation steps in the FASP are performed on an ultrafiltration unit containing a membrane with a proper molecular cut-off (typically 3000 or 10,000 Da) [103]. The proteins are first solubilized in a strong denaturing lysis buffer containing SDS and transferred to an ultrafiltration unit. SDS and other contaminants are removed by ultrafiltration, whereas proteins are retained over the membrane (Figure 2). Reduction, alkylation, and digestion are performed on the membranes. After digestion, the peptides are separated from undigested materials via ultrafiltration and collected for peptide clean-up, fractionation, and MS analysis [104]. Compared to ISD, FASP can be used for samples containing detergents [105]. However, each centrifugal step of the FASP is time-consuming (typically 20–30 min). In addition, the performance of FASP reduces when processing low amounts of protein (such as <20 μg) [106].

The FASP method has been modified for various analytical purposes. McDowell et al. developed iFASP, which combined FASP with isobaric labeling techniques, such as tandem mass tag (TMT) and isobaric tag for relative and absolute quantitation (iTRAQ) [107]. Erde et al. reported an enhanced FASP (eFASP) workflow with the substitution of 0.2% deoxycholic acid for urea to increase the efficiency of trypsin digestion of both membrane and cytosolic proteins [108]. In an express eFASP method variant, TCEP and 4-vinyl pyridine are combined for simultaneous protein reduction and alkylation, which increases the alkylation specificity and reduces the process time [108]. Yu et al. described a FASP method adapted to 96-well filter plates (96FASP) for urine sample processing [109].

FASP has been widely used in proteomics studies. For example, a recent study reported the use of MED-FASP with trypsin and LysC to analyze human muscle biopsies [110]. The authors identified >4000 proteins in slow- and fast-twitch muscle fibers. Notably, they found 237 and 172 DEPs in slow- and fast-twitch muscle fibers, respectively, after endurance exercise training for 12 weeks. In another study, 124 pairs of tumor and non-tumor esophageal tissue samples were digested using FASP, and the resultant peptides were labeled in 25 groups in a TMT 11-plex experiment [85]. The authors identified 14,252 proteins, with 784 upregulated and 747 downregulated proteins in tumors (fold-change > 1.5, adjusted *p* < 0.01). Subsequently, they stratified the samples into two subtypes based on proteomic analysis: low-risk S1 and high-risk S2. They identified ELOA and SCAF4 as subtype signatures and constructed a subtype diagnostic and prognostic model.

In a recent study, a Resolvex A200 positive-pressure solid-phase extraction unit was combined with a Bravo liquid-handling platform to perform positive-pressure FASP in a 96-well plate [111]. The method exhibited high reproducibility (Pearson correlation coefficient: r = 0.9993) when analyzing 40 technical replicates of mouse heart tissue lysates.

Zhang et al. developed a miniaturized FASP method (micro-FASP) for processing small amounts of samples [112]. The authors used a filter with approximately 0.1 mm^2^ surface area to reduce the total reagent volume to <10 μL. This device has been used to identify 1895 and 3069 protein groups in 100 and 1000 MCF-7 cells (~10 and 100 ng protein), respectively. The authors have also developed a 96-well plate version of the micro-FASP method, which showed high reproducibility (R^2^ > 0.99) when processing *Xenopus laevis* lysate (12 replicates, 1 μg each). Similarly, Sandbaumhüter et al. developed a flexible well-plate μFASP device suitable for a small amount of protein (1 μg) [113]. The filter area for the centrifugal filter units of the conventional FASP was reduced from approximately 119 mm^2^ to 0.785 mm^2^ for the μFASP device. The authors identified ~1300 proteins from 1 μg HeLa digest. From single islets of Langerhans, with <0.4 μg protein, the μFASP identified ~450 proteins.

The MStern method involves using a 0.45 µm pore polyvinylidene fluoride (PVDF) membrane to reduce the FASP processing time [114]. This method uses vacuum for each liquid transfer, which is compatible with liquid handling systems. In a 96-well PVDF filter plate, each liquid transfer step took only 1–2 min. This method processed 89 urine samples in <3 h. Subsequent analysis identified serpin B3 and B4 as potential biomarkers for ovarian cysts, since these proteins exhibited significantly higher abundance in urine from patients with ovarian cysts than in those from patients with abdominal pain controls [114]. However, MStern has not been widely used, probably owing to the lack of individual filter devices. In addition, this method increases the number of missed cleaved peptides [114,115].

Doellinger et al. have recently developed filter-aided sample preparation by easy extraction and digestion (fa-SPEED) [101]. Similar to SPEED, fa-SPEED uses TFA for protein solubilization. Acetone is used to facilitate protein aggregation before tryptic digestion. Apart from FASP, the authors used a 0.2 µm spin filter, which decreased the centrifugation time. The total hands-on time for fa-SPEED was 22 min, excluding the digestion time. This method has the advantage of a reduced processing time and is an alternative to FASP. However, this requires further investigation.

#### 3.1.3. Bead-Based Methods: Proteomic Reactor and SP3

These methods use beads to trap proteins and separate them from other contaminants. Protein trapping mechanisms include ion-exchange chromatography, HILIC, and RP chromatography. A proteomic reactor is based on protein binding on the surface of SCX beads at a low pH in a capillary column. This method was first developed by Figey et al. [116]. Reduction and alkylation are performed within capillaries. Trypsin digestion is performed by pumping trypsin solution at pH 8 into the column. At this pH, the proteins are released from the SCX beads and digested with trypsin. Subsequently, the peptides are eluted from the capillary using an ammonium bicarbonate solution. This proteomic reactor can process samples of small amounts (~10 µg protein or even 300 mouse P19 cells). Reduction, alkylation, and digestion are performed in a small effective volume (~50 nL) [116]. The mechanisms of the proteomic reactor have been used to develop a simple and integrated spin tip-based proteomics technology (SISPROT) [23], which is discussed in Section 4.

Proteomic reactors have been applied in several studies. In a previous study, a centrifugal proteomic reactor was used to identify 945 plasma membrane proteins and 955 microsomal membrane proteins in rat hepatoma cells [117]. Tao et al. used this centrifugal proteomic reactor to investigate the neuroprotective effects of huperzine A in Alzheimer’s disease and identified 2860 proteins, of which 198 were DEPs in huperzine A-treated mouse neuroblastoma N2a cells. They found that huperzine A might protect N2a cells from β-amyloid-induced cell death via p53 downregulation [118]. Liu et al. used a proteomic reactor to analyze 12 colonic biopsies from 6 patients with colorectal cancer [81]. They identified 2620 protein groups, of which 403 were differentially expressed in cancer tissues compared with that in normal tissues. Three proteins (SOD3, PRELP, and NGAL) were validated by Western blotting and could be potential tissue biomarkers for colorectal cancer. From SCX proteomic reactor, Figey et al. developed SAX proteomic reactor combined with SAX fractionation and identified ~1100 yeast proteins [24]. They also developed a 96-well plate proteomic reactor for high-throughput applications [119].

SP3 protocol is based on the use of hydrophilic carboxylate-coated paramagnetic beads [56]. Reduced and alkylated proteins are mixed with SP3 beads in a tube for protein–bead binding. These beads are separated from the contaminants in the supernatant using a magnetic rack (Figure 2). Subsequently, the proteins are digested and the resultant peptides are eluted. This method allows integration of protein and peptide enrichment, cleanup, digestion, and chemical isotope labeling in a single tube. However, the disadvantages include bead clumping and aggregation [120].

Blankenburg et al. applied the SP3 protocol for proteomic profiling of *S. aureus*, *Streptococcus suis*, and *Legionella pneumophila* [121]. The authors identified ~1470, 1150, and 1570 proteins from 5 million *S. aureus*, *S. suis*, and *L. pneumophila* cells, respectively, which are higher than the results obtained by ISD. Paulo et al. combined SP3 with TMT labeling for the proteomic profiling of two yeast species [122]. They quantified 4756 and 4429 proteins, which covered 79% and 86% proteins in the search databases of *Saccharomyces cerevisiae* and *Schizosaccharomyces pombe*, respectively. In a recent study, SP3 was used to analyze gastric tissues of 40 patients with gastric cancer [123]. Patients were divided into those with or without diabetes mellitus type 2. The authors identified ~6600 protein groups, of which 37 were differentially expressed in the diabetic cohort.

Based on the SP3 protocol, Webb et al. developed a universal solid-phase protein preparation (USP3) method for bottom-up and top-down proteomics [124]. The modifications of USP3 are the use of TCEP and CAA for simultaneous reduction and alkylation, as well as TFA to hydrolyze DNA and RNA. The advantages of this USP3 method for bottom-up proteomics are its rapid performance, scalability, high throughput, high sample handling efficiency, and low number of modification artifacts. It can also be applied to top-down proteomics [124].

The SP3 protocol has been automated in a 96-well format (AutoSP3) using a liquid-handling robot [125]. It reduced the sample processing time and increased the reproducibility of protein quantification. AutoSP3 identified ~1000 proteins from HeLa cell lysates (1 µg protein) and 459 proteins from 100 HeLa cells. Furthermore, it identified approximately 3600 proteins when analyzing a cohort of 51 clinical formalin-fixed paraffin-embedded (FFPE) pulmonary adenocarcinoma samples, AutoSP3. From the proteomic data, these 51 samples were grouped into 5 clusters corresponding to known histological growth patterns. The lepidic tissues were separated from other groups and contained 167 DEPs that could be used in further studies.

In addition to SCX and HILIC beads, RP beads have been used for protein entrapment in proteomics. Clark et al. developed a C4 RP resin tip (C4-tip) to analyze the human urine proteome [126]. The method identified >1000 protein groups in five samples (three technical replicates and two TMT 10-plex sets) with high reproducibility (R > 0.9). Adding 30% acetonitrile to the digestion buffer effectively increased peptide recovery and reduced the percentage of missed cleavage. This method demonstrates the applicability of RP beads in protein digestion. However, further studies are needed to evaluate this.

#### 3.1.4. Immobilized Enzymatic Digestion

Immobilized enzyme reactors (IMERs) are flow-through devices widely used in biological and chemical reactions. They have a high specific surface area, low reagent consumption, and a fast reaction rate [127]. In these devices, enzymes are adsorbed, entrapped, or covalently bonded to nanostructured materials or solid supports without losing their activity [128]. IMERs can process a small amount of protein (<1 µg) [129]. They can be directly coupled to HPLC or LC–MS/MS systems; however, this task is challenging due to the complexity of the instrumental setup.

Various IMERs have been developed for proteomic applications in the last two decades [130,131]. For example, an IMER with a matrix of graphene oxide-modified polymer microspheres has been developed for simultaneous protein digestion and ^18^O labeling [129]. It has been integrated online with 2-dimensional (2D) SCX–RPLC–MS/MS for the quantitative analysis of hepatocarcinoma ascites syngeneic cells. The authors quantified approximately 2950 proteins and identified 69 DEPs in samples with high lymph node metastasis rates. Qu et al. have developed an IMER that integrated hydrophilic interaction chromatography, a strong cation exchange precolumn, and PNGase F IMERs for N-linked glycosylation site profiling [132]. The authors applied this system to a soluble fraction extracted from rat brains and identified 196 N-linked glycosylation sites and 120 unique glycoproteins. Wei et al. developed an integrated microfluidic chip that combines reduction, alkylation, and on-chip trypsin IMERs [133]. Zhang et al. integrated IMERs, isotope dimethyl labeling (on a C18 precolumn), and two-dimensional peptide separation [134].

#### 3.1.5. Suspension-Trapping Method

S-Trap has gained much interest in proteomics since its development in 2014 by Zougman et al. [55]. The S-Trap tip comprises a quartz or borosilicate glass depth filter for protein trapping and an RP membrane compartment for peptide cleanup and fractionation. After reduction and alkylation, a binding buffer containing 90% methanol is used to precipitate proteins into fine suspensions stabilized by SDS [135]. The protein particulates are captured in the filter, whereas the detergents and other contaminants are separated (Figure 2). Finally, the digested peptides are cleaned up using the same tip and ready for LC–MS/MS analysis. Unlike FASP, which requires a long centrifugation time, each spinning step in the S-Trap protocol requires only 1 min, resulting in a total processing time of 20–30 min [55].

S-Trap devices, including microspin columns for <50 μg of protein, mini spin columns for <300 μg of protein, and 96-well plates, have been commercialized by ProtiFi [115]. In 2020, Zougman et al. modified S-Trap by replacing SDS with ammonium acetate or ammonium hydroxide [136]. After the proteins are trapped in the filters, detergent- and reduction/alkylation reagent-free flow-through samples containing small molecules suitable for metabolite profiling are collected. Therefore, this modified method is a simultaneous trapping (Si-Trap) method that integrates proteomics and metabolomics in the same sample extract. This method has identified 2655 proteins and 59 metabolites in renal tumor samples.

The performance of S-Trap and other protocols (such as ISD, FASP, and SP3) has been compared in some studies. For example, Miriam et al. analyzed the *Klebsiella pneumoniae* proteome and identified similar numbers of proteins (~1100) and peptides (~6300) using FASP and S-Trap [115]. The MStern method identified less proteins (<1000) and peptides (~6000) and had a high percentage of miscleavage. In *Spongospora subterranea*, S-Trap identified ~14- and ~10-fold higher numbers of peptides and proteins than SP3, respectively [137]. When characterizing the gut microbiota in mouse feces, S-Trap identified more protein and peptide with higher overlap in protein identification among three replicates, and had better reproducibility than FASP and SP3 [138]. Other studies have revealed that SP3 performs better than S-Trap in terms of peptide and protein identification, digestion efficiency, repeatability, and handling time [139,140].

The S-Trap method was used to analyze HEK 93 cells. The authors identified 4341 proteins, of which 243 were differentially expressed in methylmalonyl-CoA mutase enzyme-knockout HEK 293 cells as compared with those in HEK 293 wild-type cells [141]. Wojtkiewicz et al. used S-Trap to analyze human heart tissues, and identified ~4200 protein groups without fractionation and ~7000 protein groups with 10 high-pH RP fractions from 50 mg tissue [142]. S-Trap was used to analyze 36 sputum samples associated with *Mycobacterium tuberculosis* [115]. The authors identified 2465 human proteins and >400 microbial proteins, of which 49 were potential biomarkers for distinguishing patients from healthy participants.

#### 3.1.6. On-Slide Digestion

On-slide tissue digestion is a straightforward method used in matrix-assisted laser desorption/ionization imaging mass spectrometry (MALDI IMS) to analyze FFPE or fresh frozen tissue slides [143]. FFPE tissue sections are washed with xylene and ethanol to remove paraffin, rehydrated using a series of ethanol washes, and subjected to antigen retrieval in Tris−HCl (pH 8.0, 95 °C, 20 min). Fresh frozen tissue sections are subjected to serial ethanol washes to remove any contaminants. Tissue slides are reduced, alkylated, and digested by adding reagents [144]. A modified protocol has been reported for simultaneous glycomic and proteomic profiling of tissue sections [143]. From a 1.8 mm-diameter rat brain fresh frozen tissue section, the authors identified ~1200 proteins, 50 N-glycan compositions, 14 heparan sulfate disaccharides, and 11 chondroitin sulfate disaccharides.

#### 3.1.7. Enzymes for Digestion

Various enzymes, such as trypsin, Lys-C, Lys-N, Glu-C, Asp-N, and chymotrypsin, are used to digest proteins. Among them, trypsin is the most commonly used enzyme [145]. By cutting the polypeptide backbone on the C-terminal side of lysine and arginine, which are frequently present in proteins, trypsin digestion usually produces peptides with a positive charge of +2 or +3 and an ideal *m*/*z* for LC-MS/MS analysis [146]. Other enzymes target different amino acid residues. For example, Lys-C and Lys-N cut the polypeptide backbone on the C- and N-terminal sides of lysine, respectively. Glu-C and Asp-N target aspartic acid. Chymotrypsin targets hydrophobic amino acids, such as phenylalanine, tyrosine, and tryptophan [147].

#### 3.1.8. Enrichment of Post-Translational Modifications

PTMs are the covalent addition of functional groups to proteins after protein synthesis [148]. They can occur on the amino acid side chains or at the C- or N-termini of proteins. More than 400 types of PTMs have been identified, which alter the structure and functions of proteins and generate highly diversified and expanded proteomes. The most common PTMs are phosphorylation, acetylation, ubiquitination, methylation, and glycosylation [149]. These PTMs have been investigated using proteomics with specific PTM enrichment techniques [150]. In this part, we briefly mention some typical enrichment methods for phosphorylation, N-glycosylation, and acetylation.

Phosphorylation is the reversible addition of a phosphate group on serine, threonine, or tyrosine residues of proteins mediated by more than 800 protein kinases. It is considered the most common PTM in eukaryotic cells, which occur in ~30% of the whole proteome [151]. Typical methods for phosphorylation enrichment include immobilized metal affinity chromatography (IMAC), metal oxide affinity chromatography (MOAC), and polymer-based metal ion affinity capture (PolyMAC). They have been comprehensively discussed in some previous articles [152,153]. Zhuo et al. performed Ti^4+^-IMAC and SCX-HILIC fractionation for phosphoproteomic analysis of human cancer HeLa and K562 cells [35]. The authors identified 22,148 unique phosphopeptides and 4708 unique phosphoproteins in K562 cells from 3 mg K562 cell lysate and 11,980 unique phosphopeptides and 3424 phosphoproteins from 500 µg HeLa cell lysate.

N-glycosylation is the attachment of an oligosaccharide (glycan) to an amide nitrogen of an Asn residue in the sequence asparagine-X-serine/threonine, where X is any amino acid except proline. Typical N-glycosylation enrichment techniques are based on hydrazide, boronic acid, and lectin [154]. In the hydrazide-based technique, dialdehyde groups derived from the glycan moiety of glycoproteins or glycopeptides bind to the bead-immobilized hydrazide group [155]. Boronic acid can selectively bind to monosaccharides on glycan moieties that contain cis-diols. Therefore, boronic acid has been used for N-glycosylation enrichment in proteomics, such as immobilized–boronic acid (on magnetic beads) [156], on-plate boronic acid-modified gold nanoparticles [157], and boronate affinity monolithic columns [158]. A variety of lectins have been used to capture glycoproteins and glycopeptides. Each lectin can enrich glycoproteins and glycopeptides containing a specific glycan structure. Lectin-based N-glycosylation enrichment includes lectin affinity chromatography [159], lectin magnetic beads [160], and on-membrane lectin [161]. Mann et al. have developed a FASP-based N-linked glycopeptide capture method (N-glyco–FASP) to efficiently map the N-glycosylation sites of proteins [161]. They used lectins to enrich N-glycopeptides and PNGase F to deglycosylate the enriched N-glycopeptides. Mann et al. later described multiple enzymes for sample digestion with FASP (MED-FASP), which increased the identification of peptides, proteins, and PTM sites [162].

Acetylation is the addition of an acetyl group to the N-terminal peptide residue or the -NH_2_ of lysine. The N-terminal acetylation occurs in up to 80% of the cellular proteins and is considered one of the most frequent PTMs in eukaryotes [163]. Acetylation of the -NH_2_ of lysine residues involves some physiological processes, such as protein subcellular localization, protein–protein interactions, cell cycle, splicing, and nuclear transport [150]. Enrichment of acetylation is usually performed using an antibody-based method.

### 3.2. Peptide Purification

Peptide purification (or desalting) is typically performed using RP solid-phase extraction (SPE). Organic solvents (such as methanol) are used to condition a C18 SPE cartridge, followed by an equilibrium step using water acidified with 0.1% formic acid or trifluoroacetic acid [164]. Peptide mixtures are loaded into the cartridge and trapped by C18 RP beads, whereas contaminants are washed with washing solutions (water acidified with 0.1% formic acid or trifluoroacetic acid). Finally, the peptides are eluted with elution solutions (e.g., acetonitrile: water (8:2) acidified with 0.1% formic acid) and subsequently dried [165,166].

### 3.3. Peptide Fractionation

Peptide fractionation is performed to reduce the complexity of the peptide mixtures. Different types of fractionation have been reported, including size-exclusion chromatography [26], SCX [167], SAX [168], and middle- and high-pH RP LC [36]. These are orthogonal LC to the final low-pH RP LC step. RP LC separates peptides with respect to their hydrophobicity into long carbon chain resins [50], whereas SCX and SAX are based on the ionic properties of peptides [48,49]. Low-pH RP LC can also be used for peptide fractionation using C18 RP LC with heptafluorobutyric acid as the ion-pairing modifier. High-pH RP LC fractionation has been widely used in proteomic studies. Its elution solutions are generally acetonitrile and ammonium formate (pH 10.0) [36]. SCX fractionation is performed using elution solutions composed of water (0.1% formic acid) and 890 mM ammonium acetate solution (pH = 2.88) [167].

Peptide fractionation can be performed using two consecutive LC for in-depth proteomic profiling. For example, from 30 SCX-RP fractions, Betancourt et al. identified 5051 proteins from 29,843 peptides in mouse embryonic fibroblasts [169]. Spicer et al. identified >14,000 proteins from 126 RP–RP fractions in Jurkat cells [36]. Xu et al. developed high-pH RP LC using a polystyrene–divinylbenzene bead-packed solid-phase extraction column and then performed online SCX–RP 2D-LC–MS/MS analysis, which identified >2700 proteins from 36 RP-SCX fractions in hepatocellular carcinoma and normal liver tissues [170].

## 4. Integration of Protein Digestion, Peptide Clean-Up, and Fractionation

Integration of protein digestion, peptide clean-up, and fractionation in the same device is a promising approach to reduce sample loss and time [171]. These devices also process samples with low protein content. The primary principle of these devices is to combine different materials (such as C18, SCX, and SAX) in the same device and use appropriate solvents to perform all sample processing steps.

In some devices, C18 is used as the membrane for digestion. Mann et al. introduced stop-and-go extraction tips (StageTips) for multidimensional fractionation and desalting of peptides in 2006–2007 [172,173]. These devices were used to develop an in-StageTip (iST) method for integrating lysis, reduction, alkylation, protein digestion, peptide clean-up, and fractionation in the same device [99]. The iST devices consist of an enclosed tip stacked with a C18 membrane as a filter and optional SCX or SAX disks for fractionation. In the device reaction chamber, cell lysis, reduction, and alkylation are performed in one step, followed by protein digestion. An iST device packed with C18 and SCX disks allows in-device sample cleanup and SCX peptide fractionation. When applying the iST–SCX method, the authors identified 9667 proteins in six SCX fractions of HeLa cells with high reproducibility (R^2^ > 0.96) by label-free quantification [99]. The authors also developed an in-house iST 96-well device for high-throughput sample processing [99]. However, proteins can non-reversibly bind to the C18 materials in these iST devices, resulting in sample loss.

These iST devices have been used in several studies. Adahi et al. applied C18-SCX StageTip with acid- and salt-based elution methods and identified >22,000 phosphopeptides in 7 SCX fractions from HeLa cells [174]. Similar to SP3, the iST method is suitable for low amounts of starting protein. Sielaff et al. found that iST, SP3, and FASP performed similarly when processing 20 μg HeLa cell lysate (concerning the number of protein identifications and reproducibility) [106]. However, when the sample amount was reduced, the performance of FASP decreased, whereas SP3 and iST showed similar proteomic coverage. From samples of 25,000 immune cells, the number of proteins identified using SP3 was the highest (3152), followed by that using iST (2343) and FASP (109). Geyer et al. used the iST protocol in a 96-well device to process capillary blood plasma samples [175]. Plasma was depleted before performing the iST protocol. From 1 µL plasma, sample preparation took <2 h for denaturation, reduction, alkylation, short enzymatic digestion (1 h), and peptide clean-up. In a 20 min LC–MS/MS run, the authors identified 347 protein groups with high reproducibility (R^2^ =0.994) for 15 technical replicates.

A device similar to StageTip which combines micro-FASP and C18 microreactors in a single device, has recently been developed by Zhang et al. [176]. Digestion was performed in a C18 microreactor, whereas high-pH RP fractionation was performed using a C18 membrane. The authors identified ~4700 protein groups from 1 μg K562 cell lysate using this device.

In other devices, protein digestion is performed using SCX, SAX, or mixed SCX–SAX materials. For example, Tian et al. developed a rare cell proteomic reactor (RCPR) in 2011 to integrate protein preconcentration, contaminant removal, reduction, alkylation, and digestion in a capillary [177]. Cells were loaded onto an SCX monolith column for reduction, alkylation, and digestion. The column was then connected to a C18 RP column for online 2D LC–MS/MS analysis. Recently, Yang et al. have developed a three-frit mixed-mode RCPR that integrates protein preconcentration, reduction, alkylation, digestion, and peptide clean-up [178]. They identified ~3900, 3000, and 950 protein groups in 500, 100, and 10 cells, respectively, and ~2000–2600 protein groups in 500 mouse cochlear hair cells.

Chen et al. have developed a SISPROT device consisting of SCX beads and a C18 disk in a pipette tip [23]. This device integrates protein preconcentration, reduction, alkylation, digestion, and high pH RP peptide fractionation. Using this device, the authors identified 1709 and 717 proteins from 5000 and 2000 HEK 293 cells, respectively, within 1.4 h of MS without fractionation. In addition, the device identified 4293, 5304, and 6111 proteins from 20,000, 50,000, and 100,000 HEK 293 cells, respectively, with 5 high-pH RP fractions. Lin et al. used this device to process 1 μL serum without depletion and fractionation and identified >300 proteins within 50 min of MS using data-independent acquisition analysis [179].

Later, they developed SISPROT combining 3D peptide fractionation (3D-SISPROT) [34]. The device was similar to the SISPROT, except that SCX was replaced with SAX. Protein digestion was performed on a SAX disk, followed by SAX fractionation and high-pH RP fractionation. This 3D-SISPROT device was used to generate 3 SAX fractions using 3 buffer solutions at pH 12, 6, and 2. Subsequently, the peptides in these SAX fractions were further fractionated into five, three, and three high-pH RP fractions, respectively. As a result, the authors identified >8000 proteins from 11 high-pH RP fractions with a starting amount of 30 µg HEK 293 cell lysate. Subsequently, they developed a mixed-mode SISPROT using a 1:1 SCX and SAX beads [32]. From 1 mL serum, they generated 4 mixed-mode SCX–SAX fractions and 12 mixed-mode high-pH RP fractions and identified 862 proteins within 12 h of MS. They have also developed SISPROT–Ti^4+^-IMAC enrichment for phosphoproteome profiling [180] and Glyco–SISPROT for glycoproteome profiling [181].

## 5. Authors’ Outlook and Concluding Remarks

Sample preparation is the most crucial stage of a proteomic study, yet it can be time-consuming, labor-intensive, and error-prone [50]. Many methods, including on-membrane digestion (MStern, FASP, and fa-SPEED), bead-based digestion (proteomic reactor, SP3, and C4-tip), IMERs, S-Trap, and on-slide digestion, have been developed to replace ISD to reduce time, increase throughput, improve reproducibility, and integrate different steps in the process. The primary features of these methods are summarized in Table 1.

Among these methods, FASP has been the gold standard over the past decade [54,102]. However, it requires a long centrifugation time for each step and shows reduced performance at low protein amounts [106]. FASP cannot be integrated with downstream peptide cleanup and fractionation. The filter membrane of the FASP spin device can be damaged during a long centrifugation time [115]. Micro-FASP [112] and μFASP [103] have been developed to make this method applicable to samples with <1 μg protein. In addition, fa-SPEED can reduce the total hands-on time to 22 min by using a 0.2 µm spin filter [101]. Alternatively, S-Trap and SP3 have been used in many studies to replace the FASP. Their performance is comparable to or better than FASP performance [115,138].

In addition, many devices and methods have been developed to integrate the multiple steps of the sample preparation process (such as reduction, alkylation, digestion, and desalting) and peptide fractionation. SISPROT [23,32,34] and iST [99,106] have successfully integrated all the sample preparation steps in a single device. They considerably minimize sample loss and reduce the initial amount of protein. They are also applicable for deep proteome profiling with integration of peptide fractionation in the same device. Some sample preparation methods are compatible with automated sample preparation performed by liquid-handling workstations, such as FASP [109], S-Trap [115], SP3 [125], and iST [99,106]. They are applicable to high-throughput proteomics, particularly clinical proteomic studies, and have high precision and reproducibility. In addition, some methods, such as microFASP [112], μFASP [113], SP3 [56,121], S-Trap [55,115], SISPROT [23,32,34], and IMERs [127,128], are applicable to samples with low protein.

We expect that more technologies will be developed in the future. They may focus on three primary goals: (i) increasing the precision and reproducibility of the sample preparation process; (ii) integrating multiple steps of the sample preparation process and peptide fractionation, thereby reducing sample loss caused by manual transfer steps; and (iii) fully automating the sample preparation process for clinical studies. Advances in recent years have formed the basis for the future development of sample preparation processes in proteomics.

**Table 1:** Features of different protein digestion methods.

Method		Feature	Ref.
ISD	Urea-based ISD	Proteins are not separated from contaminants during reduction, alkylation, and digestion; not applicable for detergent-containing samples; sample loss; large starting amount (>100 µg protein); long digestion time; low throughput and reproducibility	[97]
	SCASP	Use cyclodextrin to remove SDS before digestion	[98]
	Simultaneous lysis, reduction, and alkylation	Use TCEP and CAA; reduce time and sample loss	[99]
	SPEED	Use pure TFA to dissolve cells and tissues without strong detergent; use tris(hydroxymethyl)-aminomethane to neutralize sample and precipitate proteins; reduce time and sample loss	[101]
In-gel digestion		Use polyacrylamide gel electrophoresis to separate proteins; cut, digest, and analyze gel spots separately; low throughput and reproducibility	[53]
On-membrane digestion	FASP	Use a membrane (3000 or 10,000 Da) to separate proteins from detergents and contaminants; on-membrane digestion; tolerant to strong detergent; long centrifugal time; reduced performance with samples of low protein amount (<20 μg)	[54,102,106]
	N-Glyco-FASP	Use lectins to enrich and PNGase F to deglycosylate N-glycopeptides	[161]
	MED-FASP	Use multiple enzymes for digestion	[162]
	iFASP	Combine FASP with TMT or iTRAQ	[107]
	eFASP	Use 0.2% deoxycholic acid instead of urea; increase efficiency of trypsin digestion	[108]
	Express eFASP	Use TCEP and 4-vinylpyridine for simultaneous reduction and alkylation	[108]
	MicroFASP	Use a filter with surface area of ∼0.1 mm^2^ to process low amount samples; applicable to samples of 100 cells or 1 μg protein	[112]
	μFASP	Use 96-well plates with small filter area (∼0.8 mm^2^) to process low amount samples; applicable to samples of 0.4 μg protein	[113]
	MStern	Use a membrane with 0.45 µm pore size to reduce processing time; high number of missed cleaved proteins	[114]
	fa-SPEED	Use pure TFA (similar to SPEED); use acetone to facilitate the protein aggregation; use 0.2 µm spin filter to reduce centrifugation time; quick hands-on time (~22 min, excluding digestion)	[101]
Bead-based digestion	Proteomic reactor	Use SCX or SAX beads to bind proteins; all steps are performed in a small volume (~50 nL) of a capillary; applicable to samples of low protein amount (<10 μg)	[24,116]
	SP3	Use hydrophilic carboxylate-coated paramagnetic beads to bind proteins; use a magnetic rack to separate proteins from contaminants; applicable to low protein amount (~100 ng); bead clumping and aggregation	[56,121]
	USP3	Use TFA to hydrolyze DNA and RNA; use TCEP and CAA; reduce time	[124]
	C4-tip	Use C4 RP resin tip to entrap proteins; use 30% acetonitrile in digestion buffer to increase peptide recovery and reduce missed cleavage percentage	[125]
IMERs		Flow-through devices contain entrapped enzymes; less reagent consumption; fast reaction rate; applicable to low protein amount (<1 μg); integration with fractionation and LC-MS/MS; complex instrumental setups	[127,128]
S-Trap		Use methanol to precipitate proteins; use a quartz or borosilicate glass depth filter to trap proteins; quick centrifugal time (~1 min per step, total process time ~20–30 min); integration with RP fractionation	[55,115]
On-slide digestion		Applicable to FFPE and fresh frozen tissue slides; suitable for MALDI IMS	[143,144]
Integrated methods	iST	Combine C18 membrane (filter) and SCX or SAX disks (fractionation); all steps are performed in a device; sample loss due to C18 material-protein binding; applicable to low protein amount (~1 µg)	[99,106]
	Micro-FASP + RPLC	Combine micro-FASP and C18 microreactor (sample preparation and fractionation); chemical solution volumes ~5 µL	[176]
	RCPR	Use SCX column for cell loading, protein reduction, alkylation, and digestion; combine the SCX column and a C18 RP column for 2D-LC	[177,178]
	SISPROT	Integrate SCX or SAX or SCX+SAX beads with C18 disks in a pipet tip for digestion and fractionation (1 or 2 dimensions); applicable to low protein amount (~1 µL serum)	[23,32,34]

2D, 2-dimensional; CAA, 2-chloroacetamide; eFASP, enhanced filter-aided sample preparation; fa-SPEED, FASP by easy extraction and digestion; FFPE, formalin-fixed paraffin-embedded; iFASP, isobaric labeling with FASP; IMERs, immobilized enzyme reactors; ISD, in-solution digestion; iST, in-StageTip; LC–MS/MS, liquid chromatography–tandem mass spectrometry; MALDI IMS, matrix-assisted laser desorption/ionization imaging mass spectrometry; MED-FASP, multiple enzyme digestion with FASP; RCPR, rare cell proteomic reactor; RP, reserved-phase; SAX, strong anion exchange chromatography; SCASP, sodium dodecyl sulfate (SDS)–cyclodextrin-assisted sample preparation; SCX, strong cation exchange chromatography; SP3, single-pot solid-phase-enhanced sample preparation; SPEED, sample preparation by easy extraction and digestion; TCEP, tris(2-carboxyethyl)phosphine; TFA, trifluoroacetic acid; TMT, tandem mass tag; iTRAQ, isobaric tag for relative and absolute quantitation; USP3, universal solid-phase protein preparation.

## Data Availability

Not applicable.

## References

[1] Nesvizhskii A.I., Vitek O., Aebersold R. (2007). Analysis and validation of proteomic data generated by tandem mass spectrometry. Nat. Methods.

[2] Bludau I., Aebersold R. (2020). Proteomic and interactomic insights into the molecular basis of cell functional diversity. Nat. Rev. Mol. Cell Biol..

[3] Han J., Agarwal A., Young J.N., Owji S., Luu Y., Poplausky D., Yassky D., Estrada Y., Ungar J., Krueger J.G. (2022). Proteomic profiling of a patient with cutaneous melanoma metastasis regression following topical contact sensitizer diphencyprone and immune checkpoint inhibitor treatment. Sci. Rep..

[4] Park M., Shin H.A., Duong V.-A., Lee H., Lew H. (2022). The Role of Extracellular Vesicles in Optic Nerve Injury: Neuroprotection and Mitochondrial Homeostasis. Cells.

[5] Li Y., Xu C., Wang B., Xu F., Ma F., Qu Y., Jiang D., Li K., Feng J., Tian S. (2022). Proteomic characterization of gastric cancer response to chemotherapy and targeted therapy reveals potential therapeutic strategies. Nat. Commun..

[6] Pedde R.D., Li H., Borchers C.H., Akbari M. (2017). Microfluidic-Mass Spectrometry Interfaces for Translational Proteomics. Trends Biotechnol..

[7] Zhang Z., Wu S., Stenoien D.L., Paša-Tolić L. (2014). High-Throughput Proteomics. Annu. Rev. Anal. Chem..

[8] Alexovič M., Urban P.L., Tabani H., Sabo J. (2020). Recent advances in robotic protein sample preparation for clinical analysis and other biomedical applications. Clin. Chim. Acta.

[9] Kuras M., Betancourt L.H., Rezeli M., Rodriguez J., Szasz M., Zhou Q., Miliotis T., Andersson R., Marko-Varga G. (2019). Assessing Automated Sample Preparation Technologies for High-Throughput Proteomics of Frozen Well Characterized Tissues from Swedish Biobanks. J. Proteome Res..

[10] Fu Q., Kowalski M.P., Mastali M., Parker S.J., Sobhani K., van den Broek I., Hunter C.L., Van Eyk J.E. (2018). Highly Reproducible Automated Proteomics Sample Preparation Workflow for Quantitative Mass Spectrometry. J. Proteome Res..

[11] Brodbelt J.S. (2022). Deciphering combinatorial post-translational modifications by top-down mass spectrometry. Curr. Opin. Chem. Biol..

[12] Lai Y.H., Wang Y.S. (2022). Advances in high-resolution mass spectrometry techniques for analysis of high mass-to-charge ions. Mass Spectrom. Rev..

[13] McCool E.N., Lubeckyj R.A., Shen X., Chen D., Kou Q., Liu X., Sun L. (2018). Deep Top-Down Proteomics Using Capillary Zone Electrophoresis-Tandem Mass Spectrometry: Identification of 5700 Proteoforms from the Escherichia coli Proteome. Anal. Chem..

[14] Miller R.M., Smith L.M. (2023). Overview and considerations in bottom-up proteomics. Analyst.

[15] Duong V.-A., Park J.-M., Lee H. (2020). Review of Three-Dimensional Liquid Chromatography Platforms for Bottom-Up Proteomics. Int. J. Mol. Sci..

[16] Duong V.-A., Park J.-M., Lim H.-J., Lee H. (2021). Proteomics in Forensic Analysis: Applications for Human Samples. Appl. Sci..

[17] Liang Y., Zhang L., Zhang Y. (2023). Chromatographic separation of peptides and proteins for characterization of proteomes. Chem. Commun..

[18] Nickerson J.L., Baghalabadi V., Rajendran S.R.C.K., Jakubec P.J., Said H., McMillen T.S., Dang Z., Doucette A.A. (2021). Recent advances in top-down proteome sample processing ahead of MS analysis. Mass Spectrom. Rev..

[19] Woldmar N., Schwendenwein A., Kuras M., Szeitz B., Boettiger K., Tisza A., László V., Reiniger L., Bagó A.G., Szállási Z. (2023). Proteomic analysis of brain metastatic lung adenocarcinoma reveals intertumoral heterogeneity and specific alterations associated with the timing of brain metastases. ESMO Open..

[20] Balotf S., Wilson R., Tegg R.S., Nichols D.S., Wilson C.R. (2022). Shotgun Proteomics as a Powerful Tool for the Study of the Proteomes of Plants, Their Pathogens, and Plant&ndash;Pathogen Interactions. Proteomes.

[21] Lenčo J., Jadeja S., Naplekov D.K., Krokhin O.V., Khalikova M.A., Chocholouš P., Urban J., Broeckhoven K., Nováková L., Švec F. (2022). Reversed-Phase Liquid Chromatography of Peptides for Bottom-Up Proteomics: A Tutorial. J. Proteome Res..

[22] Zhu M.-Z., Li N., Wang Y.-T., Liu N., Guo M.-Q., Sun B.-q., Zhou H., Liu L., Wu J.-L. (2017). Acid/Salt/pH Gradient Improved Resolution and Sensitivity in Proteomics Study Using 2D SCX-RP LC–MS. J. Proteome Res..

[23] Chen W., Wang S., Adhikari S., Deng Z., Wang L., Chen L., Ke M., Yang P., Tian R. (2016). Simple and Integrated Spintip-Based Technology Applied for Deep Proteome Profiling. Anal. Chem..

[24] Zhou H., Hou W., Lambert J.-P., Tian R., Figeys D. (2010). Analysis of low-abundance proteins using the proteomic reactor with pH fractionation. Talanta.

[25] Lee H.-J., Na K., Kwon M.-S., Park T., Kim K.S., Kim H., Paik Y.-K. (2011). A new versatile peptide-based size exclusion chromatography platform for global profiling and quantitation of candidate biomarkers in hepatocellular carcinoma specimens. Proteomics.

[26] Garbis S.D., Roumeliotis T.I., Tyritzis S.I., Zorpas K.M., Pavlakis K., Constantinides C.A. (2011). A Novel Multidimensional Protein Identification Technology Approach Combining Protein Size Exclusion Prefractionation, Peptide Zwitterion−Ion Hydrophilic Interaction Chromatography, and Nano-Ultraperformance RP Chromatography/nESI-MS2 for the in-Depth Analysis of the Serum Proteome and Phosphoproteome: Application to Clinical Sera Derived from Humans with Benign Prostate Hyperplasia. Anal. Chem..

[27] Di Palma S., Boersema P.J., Heck A.J.R., Mohammed S. (2011). Zwitterionic Hydrophilic Interaction Liquid Chromatography (ZIC-HILIC and ZIC-cHILIC) Provide High Resolution Separation and Increase Sensitivity in Proteome Analysis. Anal. Chem..

[28] Cao J.-L., Wang S.-S., Hu H., He C.-W., Wan J.-B., Su H.-X., Wang Y.-T., Li P. (2018). Online comprehensive two-dimensional hydrophilic interaction chromatography×reversed-phase liquid chromatography coupled with hybrid linear ion trap Orbitrap mass spectrometry for the analysis of phenolic acids in Salvia miltiorrhiza. J. Chromatogr. A.

[29] Dou M., Tsai C.-F., Piehowski P.D., Wang Y., Fillmore T.L., Zhao R., Moore R.J., Zhang P., Qian W.-J., Smith R.D. (2019). Automated Nanoflow Two-Dimensional Reversed-Phase Liquid Chromatography System Enables In-Depth Proteome and Phosphoproteome Profiling of Nanoscale Samples. Anal. Chem..

[30] Dou M., Zhu Y., Liyu A., Liang Y., Chen J., Piehowski P.D., Xu K., Zhao R., Moore R.J., Atkinson M.A. (2018). Nanowell-mediated two-dimensional liquid chromatography enables deep proteome profiling of <1000 mammalian cells. Chem. Sci..

[31] Zhao P., Schulz T.C., Sherrer E.S., Weatherly D.B., Robins A.J., Wells L. (2015). The human embryonic stem cell proteome revealed by multidimensional fractionation followed by tandem mass spectrometry. Proteomics.

[32] Xue L., Lin L., Zhou W., Chen W., Tang J., Sun X., Huang P., Tian R. (2018). Mixed-mode ion exchange-based integrated proteomics technology for fast and deep plasma proteome profiling. J. Chromatogr. A.

[33] Duong V.-A., Nam O., Jin E., Park J.-M., Lee H. (2021). Discovery of Post-Translational Modifications in Emiliania huxleyi. Molecules.

[34] Chen W., Adhikari S., Chen L., Lin L., Li H., Luo S., Yang P., Tian R. (2017). 3D-SISPROT: A simple and integrated spintip-based protein digestion and three-dimensional peptide fractionation technology for deep proteome profiling. J. Chromatogr. A.

[35] Zhou H., Di Palma S., Preisinger C., Peng M., Polat A.N., Heck A.J.R., Mohammed S. (2013). Toward a Comprehensive Characterization of a Human Cancer Cell Phosphoproteome. J. Proteome Res..

[36] Spicer V., Ezzati P., Neustaeter H., Beavis R.C., Wilkins J.A., Krokhin O.V. (2016). 3D HPLC-MS with Reversed-Phase Separation Functionality in All Three Dimensions for Large-Scale Bottom-Up Proteomics and Peptide Retention Data Collection. Anal. Chem..

[37] Kim M.-S., Pinto S.M., Getnet D., Nirujogi R.S., Manda S.S., Chaerkady R., Madugundu A.K., Kelkar D.S., Isserlin R., Jain S. (2014). A draft map of the human proteome. Nature.

[38] Wilhelm M., Schlegl J., Hahne H., Gholami A.M., Lieberenz M., Savitski M.M., Ziegler E., Butzmann L., Gessulat S., Marx H. (2014). Mass-spectrometry-based draft of the human proteome. Nature.

[39] Han X., Aslanian A., Yates J.R. (2008). Mass spectrometry for proteomics. Curr. Opin. Chem. Biol..

[40] Woods A.G., Sokolowska I., Ngounou Wetie A.G., Channaveerappa D., Dupree E.J., Jayathirtha M., Aslebagh R., Wormwood K.L., Darie C.C., Woods A.G., Darie C.C. (2019). Mass Spectrometry for Proteomics-Based Investigation. Advancements of Mass Spectrometry in Biomedical Research.

[41] Schessner J.P., Voytik E., Bludau I. (2022). A practical guide to interpreting and generating bottom-up proteomics data visualizations. Proteomics.

[42] Craig R., Beavis R.C. (2004). TANDEM: Matching proteins with tandem mass spectra. Bioinformatics.

[43] Perkins D.N., Pappin D.J.C., Creasy D.M., Cottrell J.S. (1999). Probability-based protein identification by searching sequence databases using mass spectrometry data. Electrophoresis.

[44] Eng J.K., McCormack A.L., Yates J.R. (1994). An approach to correlate tandem mass spectral data of peptides with amino acid sequences in a protein database. J. Am. Soc. Mass Spectrom..

[45] Yuan J., Zhang R., Yang Z., Lee J., Liu Y., Tian J., Qin X., Ren Z., Ding H., Chen Q. (2013). Comparative Effectiveness and Safety of Oral Phosphodiesterase Type 5 Inhibitors for Erectile Dysfunction: A Systematic Review and Network Meta-analysis. Eur. Urol..

[46] Cox J., Mann M. (2008). MaxQuant enables high peptide identification rates, individualized p.p.b.-range mass accuracies and proteome-wide protein quantification. Nat. Biotechnol..

[47] Bern M., Kil Y.J., Becker C. (2012). Byonic: Advanced Peptide and Protein Identification Software. Curr. Protoc. Bioinform..

[48] Kong A.T., Leprevost F.V., Avtonomov D.M., Mellacheruvu D., Nesvizhskii A.I. (2017). MSFragger: Ultrafast and comprehensive peptide identification in mass spectrometry–based proteomics. Nat. Methods.

[49] Chi H., Liu C., Yang H., Zeng W.-F., Wu L., Zhou W.-J., Wang R.-M., Niu X.-N., Ding Y.-H., Zhang Y. (2018). Comprehensive identification of peptides in tandem mass spectra using an efficient open search engine. Nat. Biotechnol..

[50] Danko K., Lukasheva E., Zhukov V.A., Zgoda V., Frolov A. (2022). Detergent-Assisted Protein Digestion&mdash;On the Way to Avoid the Key Bottleneck of Shotgun Bottom-Up Proteomics. Int. J. Mol. Sci..

[51] Merkley E.D., Kaiser B.L.D., Kreuzer H. (2019). A Proteomics Tutorial. Applications in Forensic Proteomics: Protein Identification and Profiling.

[52] Yang Z., Sun L. (2021). Recent technical progress in sample preparation and liquid-phase separation-mass spectrometry for proteomic analysis of mass-limited samples. Anal. Methods.

[53] Shevchenko A., Tomas H., Havli J., Olsen J.V., Mann M. (2006). In-gel digestion for mass spectrometric characterization of proteins and proteomes. Nat. Protoc..

[54] Wiśniewski J.R., Zougman A., Nagaraj N., Mann M. (2009). Universal sample preparation method for proteome analysis. Nat. Methods.

[55] Zougman A., Selby P.J., Banks R.E. (2014). Suspension trapping (STrap) sample preparation method for bottom-up proteomics analysis. PROTEOMICS.

[56] Hughes C.S., Foehr S., Garfield D.A., Furlong E.E., Steinmetz L.M., Krijgsveld J. (2014). Ultrasensitive proteome analysis using paramagnetic bead technology. Mol. Syst. Biol..

[57] Alexovič M., Sabo J., Longuespée R. (2021). Microproteomic sample preparation. Proteomics.

[58] Zhu Y., Piehowski P.D., Zhao R., Chen J., Shen Y., Moore R.J., Shukla A.K., Petyuk V.A., Campbell-Thompson M., Mathews C.E. (2018). Nanodroplet processing platform for deep and quantitative proteome profiling of 10–100 mammalian cells. Nat. Commun..

[59] Li Z.-Y., Huang M., Wang X.-K., Zhu Y., Li J.-S., Wong C.C.L., Fang Q. (2018). Nanoliter-Scale Oil-Air-Droplet Chip-Based Single Cell Proteomic Analysis. Anal. Chem..

[60] Shao X., Wang X., Guan S., Lin H., Yan G., Gao M., Deng C., Zhang X. (2018). Integrated Proteome Analysis Device for Fast Single-Cell Protein Profiling. Anal. Chem..

[61] Lamanna J., Scott E.Y., Edwards H.S., Chamberlain M.D., Dryden M.D.M., Peng J., Mair B., Lee A., Chan C., Sklavounos A.A. (2020). Digital microfluidic isolation of single cells for -Omics. Nat. Commun..

[62] Senavirathna L., Ma C., Chen R., Pan S. (2022). Spectral Library-Based Single-Cell Proteomics Resolves Cellular Heterogeneity. Cells.

[63] Kwon D., Park J.-M., Duong V.-A., Hong S.-J., Cho B.-K., Lee C.-G., Choi H.-K., Kim D.-M., Lee H. (2020). Comparative Proteomic Profiling of Marine and Freshwater Synechocystis Strains Using Liquid Chromatography-Tandem Mass Spectrometry. J. Mar. Sci. Eng..

[64] Havanapan P.-o., Thongboonkerd V. (2009). Are Protease Inhibitors Required for Gel-Based Proteomics of Kidney and Urine?. J. Proteome Res..

[65] Thingholm T.E., Larsen M.R., Ingrell C.R., Kassem M., Jensen O.N. (2008). TiO2-Based Phosphoproteomic Analysis of the Plasma Membrane and the Effects of Phosphatase Inhibitor Treatment. J. Proteome Res..

[66] Yun G., Park J.-M., Duong V.-A., Mok J.-H., Jeon J., Nam O., Lee J., Jin E., Lee H. (2020). Proteomic Profiling of Emiliania huxleyi Using a Three-Dimensional Separation Method Combined with Tandem Mass Spectrometry. Molecules.

[67] Santa C., Anjo S.I., Manadas B. (2016). Protein precipitation of diluted samples in SDS-containing buffer with acetone leads to higher protein recovery and reproducibility in comparison with TCA/acetone approach. Proteomics.

[68] Evans D.R.H., Romero J.K., Westoby M., Burgess R.R., Deutscher M.P. (2009). Chapter 9 Concentration of Proteins and Removal of Solutes. Methods in Enzymology.

[69] Koh K., Park M., Bae E.S., Duong V.-A., Park J.-M., Lee H., Lew H. (2020). UBA2 activates Wnt/β-catenin signaling pathway during protection of R28 retinal precursor cells from hypoxia by extracellular vesicles derived from placental mesenchymal stem cells. Stem Cell Res. Ther..

[70] Van-An D., Jeeyun A., Na-Young H., Jong-Moon P., Jeong-Hun M., Tae Wan K., Hookeun L. (2020). Proteomic Analysis of the Vitreous Body in Proliferative and Non-Proliferative Diabetic Retinopathy. Curr. Proteom..

[71] Olszowy P., Buszewski B. (2014). Urine sample preparation for proteomic analysis. J. Sep. Sci..

[72] Jesus J.R., Santos H.M., López-Fernández H., Lodeiro C., Arruda M.A.Z., Capelo J.L. (2018). Ultrasonic-based membrane aided sample preparation of urine proteomes. Talanta.

[73] Cao X., Sandberg A., Araújo J.E., Cvetkovski F., Berglund E., Eriksson L.E., Pernemalm M. (2021). Evaluation of Spin Columns for Human Plasma Depletion to Facilitate MS-Based Proteomics Analysis of Plasma. J. Proteome Res..

[74] Nanjappa V., Thomas J.K., Marimuthu A., Muthusamy B., Radhakrishnan A., Sharma R., Ahmad Khan A., Balakrishnan L., Sahasrabuddhe N.A., Kumar S. (2013). Plasma Proteome Database as a resource for proteomics research: 2014 update. Nucleic Acids Res..

[75] Pringels L., Broeckx V., Boonen K., Landuyt B., Schoofs L. (2018). Abundant plasma protein depletion using ammonium sulfate precipitation and Protein A affinity chromatography. J. Chromatogr. B.

[76] Beer L.A., Ky B., Barnhart K.T., Speicher D.W., Greening D.W., Simpson R.J. (2017). In-Depth, Reproducible Analysis of Human Plasma Using IgY 14 and SuperMix Immunodepletion. Serum/Plasma Proteomics: Methods and Protocols.

[77] Wu C., Duan J., Liu T., Smith R.D., Qian W.-J. (2016). Contributions of immunoaffinity chromatography to deep proteome profiling of human biofluids. J. Chromatogr. B Anal. Technol. Biomed. Life Sci..

[78] Blume J.E., Manning W.C., Troiano G., Hornburg D., Figa M., Hesterberg L., Platt T.L., Zhao X., Cuaresma R.A., Everley P.A. (2020). Rapid, deep and precise profiling of the plasma proteome with multi-nanoparticle protein corona. Nat. Commun..

[79] Tu C., Rudnick P.A., Martinez M.Y., Cheek K.L., Stein S.E., Slebos R.J.C., Liebler D.C. (2010). Depletion of abundant plasma proteins and limitations of plasma proteomics. J. Proteome Res..

[80] Oliveira B.P., Buzalaf M.A.R., Silva N.C., Ventura T.M.O., Toniolo J., Rodrigues J.A. (2022). Saliva proteomic profile of early childhood caries and caries-free children. Acta Odontol. Scand..

[81] Liu X., Xu Y., Meng Q., Zheng Q., Wu J., Wang C., Jia W., Figeys D., Chang Y., Zhou H. (2016). Proteomic analysis of minute amount of colonic biopsies by enteroscopy sampling. Biochem. Biophys. Res. Commun..

[82] Toni M., Angiulli E., Miccoli G., Cioni C., Alleva E., Frabetti F., Pizzetti F., Grassi Scalvini F., Nonnis S., Negri A. (2019). Environmental temperature variation affects brain protein expression and cognitive abilities in adult zebrafish (Danio rerio): A proteomic and behavioural study. J. Proteom..

[83] Smith K.M., Xu Y. (2012). Tissue sample preparation in bioanalytical assays. Bioanalysis.

[84] Yagi R., Masuda T., Ogata S., Mori A., Ito S., Ohtsuki S. (2020). Proteomic Evaluation of Plasma Membrane Fraction Prepared from a Mouse Liver and Kidney Using a Bead Homogenizer: Enrichment of Drug-Related Transporter Proteins. Mol. Pharm..

[85] Liu W., Xie L., He Y.-H., Wu Z.-Y., Liu L.-X., Bai X.-F., Deng D.-X., Xu X.-E., Liao L.-D., Lin W. (2021). Large-scale and high-resolution mass spectrometry-based proteomics profiling defines molecular subtypes of esophageal cancer for therapeutic targeting. Nat. Commun..

[86] Dubacq S. (2016). Performing efficient sample preparation with hard tumor tissue: Precellys^®^ bead-beating homogenizer solution. Nat. Methods.

[87] Prieto D.A., Whitely G., Johann D.J., Blonder J., Murray G.I. (2018). Protocol for the Analysis of Laser Capture Microdissected Fresh-Frozen Tissue Homogenates by Silver-Stained 1D SDS-PAGE. Laser Capture Microdissection: Methods and Protocols.

[88] Mertins P., Tang L.C., Krug K., Clark D.J., Gritsenko M.A., Chen L., Clauser K.R., Clauss T.R., Shah P., Gillette M.A. (2018). Reproducible workflow for multiplexed deep-scale proteome and phosphoproteome analysis of tumor tissues by liquid chromatography–mass spectrometry. Nat. Protoc..

[89] Valdés-López O., Batek J., Gomez-Hernandez N., Nguyen C.T., Isidra-Arellano M.C., Zhang N., Joshi T., Xu D., Hixson K.K., Weitz K.K. (2016). Soybean Roots Grown under Heat Stress Show Global Changes in Their Transcriptional and Proteomic Profiles. Front. Plant Sci..

[90] Guyuron B., Yohannes E., Miller R., Chim H., Reed D., Chance M.R. (2014). Electron Microscopic and Proteomic Comparison of Terminal Branches of the Trigeminal Nerve in Patients with and without Migraine Headaches. Plast. Reconstr. Surg..

[91] Cai X., Xue Z., Wu C., Sun R., Qian L., Yue L., Ge W., Yi X., Liu W., Chen C. (2022). High-throughput proteomic sample preparation using pressure cycling technology. Nat. Protoc..

[92] Bradford M.M. (1976). A rapid and sensitive method for the quantitation of microgram quantities of protein utilizing the principle of protein-dye binding. Anal. Biochem..

[93] Donnell A.M., Lewis S., Abraham S., Subramanian K., Figueroa J.L., Deepe G.S., Vonderheide A.P. (2017). Investigation of an optimal cell lysis method for the study of the zinc metalloproteome of Histoplasma capsulatum. Anal. Bioanal. Chem..

[94] Smith P.K., Krohn R.I., Hermanson G.T., Mallia A.K., Gartner F.H., Provenzano M.D., Fujimoto E.K., Goeke N.M., Olson B.J., Klenk D.C. (1985). Measurement of protein using bicinchoninic acid. Anal. Biochem..

[95] Legler G., Müller-Platz C.M., Mentges-Hettkamp M., Pflieger G., Jülich E. (1985). On the chemical basis of the Lowry protein determination. Anal. Biochem..

[96] Oswald E.S., Brown L.M., Bulinski J.C., Hung C.T. (2011). Label-Free Protein Profiling of Adipose-Derived Human Stem Cells under Hyperosmotic Treatment. J. Proteome Res..

[97] Yu Y.-Q., Gilar M., Lee P.J., Bouvier E.S.P., Gebler J.C. (2003). Enzyme-Friendly, Mass Spectrometry-Compatible Surfactant for In-Solution Enzymatic Digestion of Proteins. Anal. Chem..

[98] Gan G., Xu X., Chen X., Zhang X.-F., Wang J., Zhong C.-Q. (2021). SCASP: A Simple and Robust SDS-Aided Sample Preparation Method for Proteomic Research. Mol. Cell. Proteom..

[99] Kulak N.A., Pichler G., Paron I., Nagaraj N., Mann M. (2014). Minimal, encapsulated proteomic-sample processing applied to copy-number estimation in eukaryotic cells. Nat. Methods.

[100] Gebreyesus S.T., Siyal A.A., Kitata R.B., Chen E.S.-W., Enkhbayar B., Angata T., Lin K.-I., Chen Y.-J., Tu H.-L. (2022). Streamlined single-cell proteomics by an integrated microfluidic chip and data-independent acquisition mass spectrometry. Nat. Commun..

[101] Doellinger J., Schneider A., Hoeller M., Lasch P. (2020). Sample Preparation by Easy Extraction and Digestion (SPEED)—A Universal, Rapid, and Detergent-free Protocol for Proteomics Based on Acid Extraction. Mol. Amp; Cell. Proteom..

[102] Manza L.L., Stamer S.L., Ham A.-J.L., Codreanu S.G., Liebler D.C. (2005). Sample preparation and digestion for proteomic analyses using spin filters. Proteomics.

[103] Wither M.J., Hansen K.C., Reisz J.A. (2016). Mass Spectrometry-Based Bottom-Up Proteomics: Sample Preparation, LC-MS/MS Analysis, and Database Query Strategies. Curr. Protoc. Protein Sci..

[104] Wiśniewski J.R. (2019). Filter Aided Sample Preparation—A tutorial. Anal. Chim. Acta.

[105] Davalieva K., Kiprijanovska S., Dimovski A., Rosoklija G., Dwork A.J. (2021). Comparative evaluation of two methods for LC-MS/MS proteomic analysis of formalin fixed and paraffin embedded tissues. J. Proteom..

[106] Sielaff M., Kuharev J., Bohn T., Hahlbrock J., Bopp T., Tenzer S., Distler U. (2017). Evaluation of FASP, SP3, and iST Protocols for Proteomic Sample Preparation in the Low Microgram Range. J. Proteome Res..

[107] McDowell G.S., Gaun A., Steen H. (2013). iFASP: Combining Isobaric Mass Tagging with Filter-Aided Sample Preparation. J. Proteome Res..

[108] Erde J., Loo R.R.O., Loo J.A. (2014). Enhanced FASP (eFASP) to Increase Proteome Coverage and Sample Recovery for Quantitative Proteomic Experiments. J. Proteome Res..

[109] Yu Y., Suh M.-J., Sikorski P., Kwon K., Nelson K.E., Pieper R. (2014). Urine Sample Preparation in 96-Well Filter Plates for Quantitative Clinical Proteomics. Anal. Chem..

[110] Deshmukh A.S., Steenberg D.E., Hostrup M., Birk J.B., Larsen J.K., Santos A., Kjøbsted R., Hingst J.R., Schéele C.C., Murgia M. (2021). Deep muscle-proteomic analysis of freeze-dried human muscle biopsies reveals fiber type-specific adaptations to exercise training. Nat. Commun..

[111] Loroch S., Kopczynski D., Schneider A.C., Schumbrutzki C., Feldmann I., Panagiotidis E., Reinders Y., Sakson R., Solari F.A., Vening A. (2022). Toward Zero Variance in Proteomics Sample Preparation: Positive-Pressure FASP in 96-Well Format (PF96) Enables Highly Reproducible, Time- and Cost-Efficient Analysis of Sample Cohorts. J. Proteome Res..

[112] Zhang Z., Dubiak K.M., Huber P.W., Dovichi N.J. (2020). Miniaturized Filter-Aided Sample Preparation (MICRO-FASP) Method for High Throughput, Ultrasensitive Proteomics Sample Preparation Reveals Proteome Asymmetry in Xenopus laevis Embryos. Anal. Chem..

[113] Sandbaumhüter F.A., Nezhyva M., Eriksson O., Engberg A., Kreuger J., Andrén P.E., Jansson E.T. (2022). Well-Plate μFASP for Proteomic Analysis of Single Pancreatic Islets. J. Proteome Res..

[114] Berger S.T., Ahmed S., Muntel J., Cuevas Polo N., Bachur R., Kentsis A., Steen J., Steen H. (2015). MStern Blotting–High Throughput Polyvinylidene Fluoride (PVDF) Membrane-Based Proteomic Sample Preparation for 96-Well Plates*[S]. Mol. Cell. Proteom..

[115] HaileMariam M., Eguez R.V., Singh H., Bekele S., Ameni G., Pieper R., Yu Y. (2018). S-Trap, an Ultrafast Sample-Preparation Approach for Shotgun Proteomics. J. Proteome Res..

[116] Ethier M., Hou W., Duewel H.S., Figeys D. (2006). The Proteomic Reactor:  A Microfluidic Device for Processing Minute Amounts of Protein Prior to Mass Spectrometry Analysis. J. Proteome Res..

[117] Zhou H., Wang F., Wang Y., Ning Z., Hou W., Wright T.G., Sundaram M., Zhong S., Yao Z., Figeys D. (2011). Improved Recovery and Identification of Membrane Proteins from Rat Hepatic Cells using a Centrifugal Proteomic Reactor*. Mol. Cell. Proteom..

[118] Tao Y., Fang L., Yang Y., Jiang H., Yang H., Zhang H., Zhou H. (2013). Quantitative proteomic analysis reveals the neuroprotective effects of huperzine A for amyloid beta treated neuroblastoma N2a cells. Proteomics.

[119] Hou W., Ethier M., Smith J.C., Sheng Y., Figeys D. (2007). Multiplexed Proteomic Reactor for the Processing of Proteomic Samples. Anal. Chem..

[120] Hughes C.S., Moggridge S., Müller T., Sorensen P.H., Morin G.B., Krijgsveld J. (2019). Single-pot, solid-phase-enhanced sample preparation for proteomics experiments. Nat. Protoc..

[121] Blankenburg S., Hentschker C., Nagel A., Hildebrandt P., Michalik S., Dittmar D., Surmann K., Völker U. (2019). Improving Proteome Coverage for Small Sample Amounts: An Advanced Method for Proteomics Approaches with Low Bacterial Cell Numbers. Proteomics.

[122] Paulo J.A., Navarrete-Perea J., Gygi S.P. (2020). Multiplexed proteome profiling of carbon source perturbations in two yeast species with SL-SP3-TMT. J. Proteom..

[123] Osório H., Silva C., Ferreira M., Gullo I., Máximo V., Barros R., Mendonça F., Oliveira C., Carneiro F. (2021). Proteomics Analysis of Gastric Cancer Patients with Diabetes Mellitus. J. Clin. Med..

[124] Dagley L.F., Infusini G., Larsen R.H., Sandow J.J., Webb A.I. (2019). Universal Solid-Phase Protein Preparation (USP3) for Bottom-up and Top-down Proteomics. J. Proteome Res..

[125] Müller T., Kalxdorf M., Longuespée R., Kazdal D.N., Stenzinger A., Krijgsveld J. (2020). Automated sample preparation with SP3 for low-input clinical proteomics. Mol. Syst. Biol..

[126] Clark D.J., Hu Y., Schnaubelt M., Fu Y., Ponce S., Chen S.-Y., Zhou Y., Shah P., Zhang H. (2019). Simple Tip-Based Sample Processing Method for Urinary Proteomic Analysis. Anal. Chem..

[127] Wouters B., Currivan S.A., Abdulhussain N., Hankemeier T., Schoenmakers P.J. (2021). Immobilized-enzyme reactors integrated into analytical platforms: Recent advances and challenges. TrAC Trends Anal. Chem..

[128] Yamaguchi H., Miyazaki M. (2013). Enzyme-immobilized reactors for rapid and efficient sample preparation in MS-based proteomic studies. Proteomics.

[129] Yuan H., Zhang S., Zhao B., Weng Y., Zhu X., Li S., Zhang L., Zhang Y. (2017). Enzymatic Reactor with Trypsin Immobilized on Graphene Oxide Modified Polymer Microspheres To Achieve Automated Proteome Quantification. Anal. Chem..

[130] Ma J., Zhang L., Liang Z., Shan Y., Zhang Y. (2011). Immobilized enzyme reactors in proteomics. TrAC Trends Anal. Chem..

[131] Nagy C., Szabo R., Gaspar A. (2022). Microfluidic Immobilized Enzymatic Reactors for Proteomic Analyses&mdash;Recent Developments and Trends (2017–2021). Micromachines.

[132] Qu Y., Xia S., Yuan H., Wu Q., Li M., Zou L., Zhang L., Liang Z., Zhang Y. (2011). Integrated Sample Pretreatment System for N-Linked Glycosylation Site Profiling with Combination of Hydrophilic Interaction Chromatography and PNGase F Immobilized Enzymatic Reactor via a Strong Cation Exchange Precolumn. Anal. Chem..

[133] Wei Z., Fan P., Jiao Y., Wang Y., Huang Y., Liu Z. (2020). Integrated microfluidic chip for on-line proteome analysis with combination of denaturing and rapid digestion of protein. Anal. Chim. Acta.

[134] Zhang S., Yuan H., Zhao B., Zhang L., Zhang Y. (2018). Integrated platform with combination of on-line protein digestion, isotope dimethyl labeling and multidimensional peptide separation for high-throughput proteome quantification. Anal. Chim. Acta.

[135] Duong V.-A., Park J.-M., Lee H. (2022). A review of suspension trapping digestion method in bottom-up proteomics. J. Sep. Sci..

[136] Zougman A., Wilson J.P., Roberts L.D., Banks R.E. (2020). Detergent-Free Simultaneous Sample Preparation Method for Proteomics and Metabolomics. J. Proteome Res..

[137] Balotf S., Wilson R., Tegg R.S., Nichols D.S., Wilson C.R. (2020). Optimisation of Sporosori Purification and Protein Extraction Techniques for the Biotrophic Protozoan Plant Pathogen Spongospora subterranea. Molecules.

[138] Baniasad M., Kim Y., Shaffer M., Sabag-Daigle A., Leleiwi I., Daly R.A., Ahmer B.M.M., Wrighton K.C., Wysocki V.H. (2022). Optimization of proteomics sample preparation for identification of host and bacterial proteins in mouse feces. Anal. Bioanal. Chem..

[139] Hayoun K., Gouveia D., Grenga L., Pible O., Armengaud J., Alpha-Bazin B. (2019). Evaluation of Sample Preparation Methods for Fast Proteotyping of Microorganisms by Tandem Mass Spectrometry. Front. Microbiol..

[140] Mikulášek K., Konečná H., Potěšil D., Holánková R., Havliš J., Zdráhal Z. (2021). SP3 Protocol for Proteomic Plant Sample Preparation Prior LC-MS/MS. Front. Plant Sci..

[141] Costanzo M., Caterino M., Cevenini A., Jung V., Chhuon C., Lipecka J., Fedele R., Guerrera I.C., Ruoppolo M. (2020). Dataset of a comparative proteomics experiment in a methylmalonyl-CoA mutase knockout HEK 293 cell model. Data Brief.

[142] Wojtkiewicz M., Berg Luecke L., Kelly M.I., Gundry R.L. (2021). Facile Preparation of Peptides for Mass Spectrometry Analysis in Bottom-Up Proteomics Workflows. Curr. Protoc..

[143] Raghunathan R., Sethi M.K., Zaia J. (2019). On-slide tissue digestion for mass spectrometry based glycomic and proteomic profiling. MethodsX.

[144] Judd A.M., Gutierrez D.B., Moore J.L., Patterson N.H., Yang J., Romer C.E., Norris J.L., Caprioli R.M. (2019). A recommended and verified procedure for in situ tryptic digestion of formalin-fixed paraffin-embedded tissues for analysis by matrix-assisted laser desorption/ionization imaging mass spectrometry. J. Mass Spectrom..

[145] Olsen J.V., Ong S.-E., Mann M. (2004). Trypsin Cleaves Exclusively C-terminal to Arginine and Lysine Residues*. Mol. Cell. Proteom..

[146] Tyers M., Mann M. (2003). From genomics to proteomics. Nature.

[147] Giansanti P., Tsiatsiani L., Low T.Y., Heck A.J.R. (2016). Six alternative proteases for mass spectrometry–based proteomics beyond trypsin. Nat. Protoc..

[148] Li W., Li F., Zhang X., Lin H.-K., Xu C. (2021). Insights into the post-translational modification and its emerging role in shaping the tumor microenvironment. Signal Transduct. Target. Ther..

[149] Ramazi S., Zahiri J. (2021). Post-translational modifications in proteins: Resources, tools and prediction methods. Database.

[150] Pieroni L., Iavarone F., Olianas A., Greco V., Desiderio C., Martelli C., Manconi B., Sanna M.T., Messana I., Castagnola M. (2020). Enrichments of post-translational modifications in proteomic studies. J. Sep. Sci..

[151] Humphrey S.J., James D.E., Mann M. (2015). Protein Phosphorylation: A Major Switch Mechanism for Metabolic Regulation. Trends Endocrinol. Metab..

[152] Fíla J., Honys D. (2012). Enrichment techniques employed in phosphoproteomics. Amino Acids.

[153] Qiu W., Evans C.A., Landels A., Pham T.K., Wright P.C. (2020). Phosphopeptide enrichment for phosphoproteomic analysis—A tutorial and review of novel materials. Anal. Chim. Acta.

[154] Ahn Y.H., Kim J.Y., Yoo J.S. (2015). Quantitative mass spectrometric analysis of glycoproteins combined with enrichment methods. Mass Spectrom. Rev..

[155] Zhang H., Li X.-j., Martin D.B., Aebersold R. (2003). Identification and quantification of N-linked glycoproteins using hydrazide chemistry, stable isotope labeling and mass spectrometry. Nat. Biotechnol..

[156] Lee J.H., Kim Y., Ha M.Y., Lee E.K., Choo J. (2005). Immobilization of aminophenylboronic acid on magnetic beads for the direct determination of glycoproteins by matrix assisted laser desorption ionization mass spectrometry. J. Am. Soc. Mass Spectrom..

[157] Tang J., Liu Y., Qi D., Yao G., Deng C., Zhang X. (2009). On-plate-selective enrichment of glycopeptides using boronic acid-modified gold nanoparticles for direct MALDI-QIT-TOF MS analysis. Proteomics.

[158] Lin Z.A., Pang J.L., Lin Y., Huang H., Cai Z.W., Zhang L., Chen G.N. (2011). Preparation and evaluation of a phenylboronate affinity monolith for selective capture of glycoproteins by capillary liquid chromatography. Analyst.

[159] Heo S.-H., Lee S.-J., Ryoo H.-M., Park J.-Y., Cho J.-Y. (2007). Identification of putative serum glycoprotein biomarkers for human lung adenocarcinoma by multilectin affinity chromatography and LC-MS/MS. Proteomics.

[160] Choi E., Loo D., Dennis J.W., O’Leary C.A., Hill M.M. (2011). High-throughput lectin magnetic bead array-coupled tandem mass spectrometry for glycoprotein biomarker discovery. Electrophoresis.

[161] Zielinska D.F., Gnad F., Wiśniewski J.R., Mann M. (2010). Precision Mapping of an In Vivo N-Glycoproteome Reveals Rigid Topological and Sequence Constraints. Cell.

[162] Wiśniewski J.R., Mann M. (2012). Consecutive Proteolytic Digestion in an Enzyme Reactor Increases Depth of Proteomic and Phosphoproteomic Analysis. Anal. Chem..

[163] Aksnes H., Van Damme P., Goris M., Starheim K.K., Marie M., Støve S.I., Hoel C., Kalvik T.V., Hole K., Glomnes N. (2015). An Organellar Nα-Acetyltransferase, Naa60, Acetylates Cytosolic N Termini of Transmembrane Proteins and Maintains Golgi Integrity. Cell Rep..

[164] Schmelter C., Funke S., Treml J., Beschnitt A., Perumal N., Manicam C., Pfeiffer N., Grus F.H. (2018). Comparison of Two Solid-Phase Extraction (SPE) Methods for the Identification and Quantification of Porcine Retinal Protein Markers by LC-MS/MS. Int. J. Mol. Sci..

[165] Shen Y., Tolić N., Masselon C., Paša-Tolić L., Camp D.G., Hixson K.K., Zhao R., Anderson G.A., Smith R.D. (2004). Ultrasensitive Proteomics Using High-Efficiency On-Line Micro-SPE-NanoLC-NanoESI MS and MS/MS. Anal. Chem..

[166] Bladergroen M.R., van der Burgt Y.E.M. (2015). Solid-phase extraction strategies to surmount body fluid sample complexity in high-throughput mass spectrometry-based proteomics. J. Anal. Methods Chem..

[167] Chen D., Shen X., Sun L. (2018). Strong cation exchange-reversed phase liquid chromatography-capillary zone electrophoresis-tandem mass spectrometry platform with high peak capacity for deep bottom-up proteomics. Anal. Chim. Acta.

[168] Boichenko A.P., Govorukhina N., van der Zee A.G.J., Bischoff R. (2013). Multidimensional separation of tryptic peptides from human serum proteins using reversed-phase, strong cation exchange, weak anion exchange, and fused-core fluorinated stationary phases. J. Sep. Sci..

[169] Betancourt L.H., De Bock P.-J., Staes A., Timmerman E., Perez-Riverol Y., Sanchez A., Besada V., Gonzalez L.J., Vandekerckhove J., Gevaert K. (2013). SCX charge state selective separation of tryptic peptides combined with 2D-RP-HPLC allows for detailed proteome mapping. J. Proteom..

[170] Xu B., Wang F., Song C., Sun Z., Cheng K., Tan Y., Wang H., Zou H. (2014). Large-Scale Proteome Quantification of Hepatocellular Carcinoma Tissues by a Three-Dimensional Liquid Chromatography Strategy Integrated with Sample Preparation. J. Proteome Res..

[171] Ye X., Tang J., Mao Y., Lu X., Yang Y., Chen W., Zhang X., Xu R., Tian R. (2019). Integrated proteomics sample preparation and fractionation: Method development and applications. TrAC Trends Anal. Chem..

[172] Ishihama Y., Rappsilber J., Mann M. (2006). Modular Stop and Go Extraction Tips with Stacked Disks for Parallel and Multidimensional Peptide Fractionation in Proteomics. J. Proteome Res..

[173] Rappsilber J., Mann M., Ishihama Y. (2007). Protocol for micro-purification, enrichment, pre-fractionation and storage of peptides for proteomics using StageTips. Nat. Protoc..

[174] Adachi J., Hashiguchi K., Nagano M., Sato M., Sato A., Fukamizu K., Ishihama Y., Tomonaga T. (2016). Improved Proteome and Phosphoproteome Analysis on a Cation Exchanger by a Combined Acid and Salt Gradient. Anal. Chem..

[175] Geyer P.E., Kulak N.A., Pichler G., Holdt L.M., Teupser D., Mann M. (2016). Plasma Proteome Profiling to Assess Human Health and Disease. Cell Syst..

[176] Zhang Z., Dovichi N.J. (2022). Seamlessly Integrated Miniaturized Filter-Aided Sample Preparation Method to Fractionation Techniques for Fast, Loss-Less, and In-Depth Proteomics Analysis of 1 μg of Cell Lysates at Low Cost. Anal. Chem..

[177] Tian R., Wang S., Elisma F., Li L., Zhou H., Wang L., Figeys D. (2011). Rare Cell Proteomic Reactor Applied to Stable Isotope Labeling by Amino Acids in Cell Culture (SILAC)-based Quantitative Proteomics Study of Human Embryonic Stem Cell Differentiation. Mol. Cell. Proteom..

[178] Yang Y., Sun S., He S., Liu C., Fu C., Tang M., Liu C., Sun Y., Lam H., Liu Z. (2023). Fully integrated on-line strategy for highly sensitive proteome profiling of 10–500 mammalian cells. Analyst.

[179] Lin L., Zheng J., Yu Q., Chen W., Xing J., Chen C., Tian R. (2018). High throughput and accurate serum proteome profiling by integrated sample preparation technology and single-run data independent mass spectrometry analysis. J. Proteom..

[180] Chen W., Chen L., Tian R. (2018). An integrated strategy for highly sensitive phosphoproteome analysis from low micrograms of protein samples. Analyst.

[181] Gao W., Li H., Liu L., Huang P., Wang Z., Chen W., Ye M., Yu X., Tian R. (2019). An integrated strategy for high-sensitive and multi-level glycoproteome analysis from low micrograms of protein samples. J. Chromatogr. A.

